# Valvular Heart Disease Epidemiology

**DOI:** 10.3390/medsci10020032

**Published:** 2022-06-15

**Authors:** John Sukumar Aluru, Adam Barsouk, Kalyan Saginala, Prashanth Rawla, Alexander Barsouk

**Affiliations:** 1 Minneapolis Heart Institute Foundation, Allina Health, Minneapolis, MN 55407, USA; john.aluru@allina.com; 2 Hillman Cancer Center, University of Pittsburgh, Pittsburgh, PA 15232, USA; adambarsouk@comcast.net; 3 Plains Regional Medical Group Internal Medicine, Clovis, NM 88101, USA; drsaginala@gmail.com; 4 Parrish Medical Center, Titusville, FL 32796, USA; 5 Allegheny Health Network, Pittsburgh, PA 15212, USA; alexbarsouk@comcast.net

**Keywords:** valvular heart disease, epidemiology, aortic valve stenotic disease, rheumatic heart disease, aortic regurgitation, tricuspid regurgitation, infective endocarditis

## Abstract

Valvular heart disease is a rapidly growing cause of global cardiovascular morbidity and mortality with diverse and evolving geographic distribution. The prevalence of rheumatic heart disease, the most common valvular heart disease (affecting approximately 41 million people), has been rising in developing nations, likely due to the expansion of the young adult population and the decrease in premature mortality that has resulted from improved access to antibiotics, microbiological testing, and echocardiography. Rheumatic heart disease has also been rising among the impoverished and, often, indigenous populations of developed nations, spurring public health initiatives that are aimed at alleviating healthcare disparities. Aortic valve stenotic disease is the most commonly occurring valvular pathology in developed nations (afflicting 9 million people worldwide) and its prevalence has been increasing with population aging and the increased prevalence of atherosclerosis. Aortic regurgitation is associated with diastolic, but not systolic, hypertension and it has likewise seen a rise in the developed world. Mitral regurgitation affects 24 million people worldwide, with great variability between and among nations. Primary mitral regurgitation arises as a consequence of myxomatous degeneration and mitral valve prolapse, which is largely due to genetic predispositions, while secondary mitral regurgitation accounts for 65% of cases and arises secondary to dilation and heart failure. Tricuspid regurgitation has become more prevalent in developed nations due to the increased usage of intracardiac pacemakers. Infective endocarditis prevalence has also grown in developed nations, likely due to population aging and the increased utilization of transcatheter valve replacement and prosthetic valves as interventions against the previously discussed valvular pathologies.

## 1. Introduction

Valvular heart disease is a leading cause of cardiovascular morbidity and mortality worldwide and the resultant disease burden is only projected to increase in the coming decades. The heart contains four valves: the tricuspid, pulmonic, mitral, and aortic. These valves prevent backward flow between the four heart chambers and maintain the pressure gradients that are necessary for the hemodynamic circulation that is essential for life. The regurgitation from or the insufficiency of the valves are secondary to valvular heart disease and both permit backward flow, resulting in an equalization of pressure that can be incompatible with cardiovascular function. Conversely, the stenosis of the valves, which is secondary to valvular heart disease, results in increased pressures behind the blockage (often resulting in cardiac remodeling) and insufficient pressure ahead of the blockage (e.g., syncope in the case of aortic stenosis) [1,2]. Understanding the geographical and temporal trends that are present in valve disease epidemiology is crucial for designing effective public health interventions for primary and secondary prevention. Global epidemiological data can be unreliable, as post-mortem analysis has revealed the true prevalence of valvular heart disease to be significantly greater than that which is clinically coded and reported [3]. Furthermore, limited access to echocardiography and even microbiological testing likely results in the severe underreporting of valvular disease in developing nations and indigenous and impoverished populations in developed nations [4].

**Figure 1:**
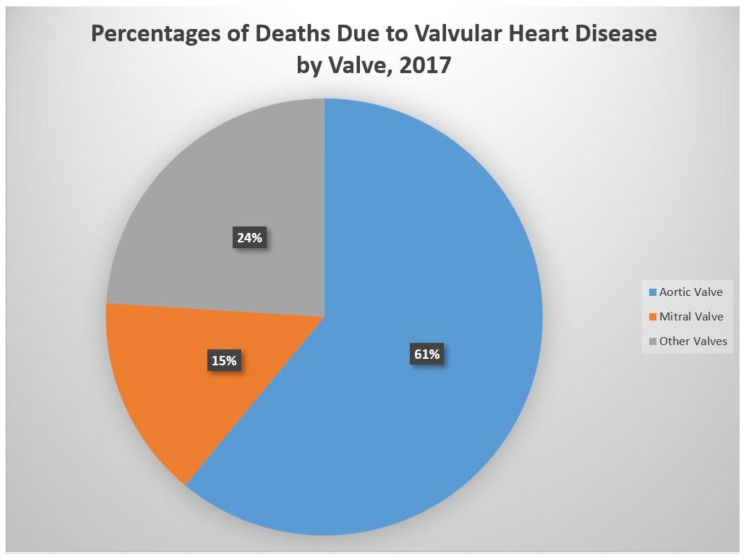
Percentages of deaths due to valvular heart disease, by valve, 2017—data obtained from CDC, Atlanta, GA, USA. Available online: https://www.cdc.gov/heartdisease/valvular_disease.htm (Accessed on 25 April 2022).

The most prevalent valve pathologies, globally, are rheumatic heart disease, aortic valve stenotic disease, mitral regurgitation, and aortic regurgitation, while in the developed world, aortic valve stenotic disease is more prevalent [5]. Diseases of the aortic valve account for 61% of all valvular heart disease deaths, while diseases of the mitral valve account for 15% (Figure 1). Diseases of the aortic valve have a well-established association with old age and chronic cardiovascular disease, while rheumatic heart disease is an infectious complication that is primarily associated with overcrowding and poor healthcare access. Although infective endocarditis is similarly bacterial in its pathogenesis, it is more common in the elderly and likewise more prevalent in developed nations [1,2]. While women account for a larger proportion of valvular heart disease cases worldwide, they have often been underrepresented in the landmark studies that have informed treatment guidelines and have reported worse postoperative outcomes [6].

Echocardiography is the gold standard for the diagnosis of valvular disease and most patients with progressive disease should be followed up with at least annually by a cardiologist. Intervention is warranted in symptomatic patients or those with diminished ventricular function, though the indications for intervention are disease-specific. Transcatheter valve replacement, which is often guided by echocardiography, has gained popularity over open-heart surgery in aortic valve replacement and mitral clip procedures and has likely improved the prognosis for certain valvular heart diseases [7].

## 2. Aortic Valve Stenotic Disease

### 2.1. Epidemiology

Aortic valve stenotic disease (AVSD) is the most prevalent valvular disease in the developed world. The predominant form of AVSD is calcific AVSD, which occurs due to the thickening and calcification of the aortic valve (i.e., “aortic stenosis”). Rarely, AVSD can also be caused by infection (post-endocarditis scarring). The normal valve area of the aortic valve is 3 cm^2^; the symptoms of aortic stenosis do not typically develop until the valve area is <1 cm^2^. The classic symptoms of AVSD include angina, dyspnea on exertion, syncope, and ultimate heart failure. A physical exam classically reveals a crescendo–decrescendo systolic murmur that is best heard at the base of the heart with radiation to the carotid arteries. Other physical exam findings include delayed carotid upstroke (pulsus parvus et tardus), sustained point of maximal impulse (PMI), and a diminished A2 sound. Cardiac auscultation has limited accuracy for the detection of valvular heart disease in asymptomatic patients and is a poor diagnostic screening tool when it is used in primary care [8].

The relative frequency of the causes of ASVD varies geographically. Worldwide, rheumatic valve disease is the most common cause of ASVD and mitral valve involvement invariably accompanies rheumatic aortic valve disease. In Europe and North America, aortic valve disease primarily occurs due to a calcific disease of a native trileaflet valve or a congenitally bicuspid valve.

A prospective population-based study of 3273 participants, including 164 subjects with ASVD, showed that the prevalence of AS increases with age. The prevalence of ASVD varied from 0.2 percent at ages 50 to 59 years, 1.3 percent at ages 60 to 69, 3.9 percent at ages 70 to 79 years, and 9.8 percent at ages 80 to 89 years [9].

**Figure 2:**
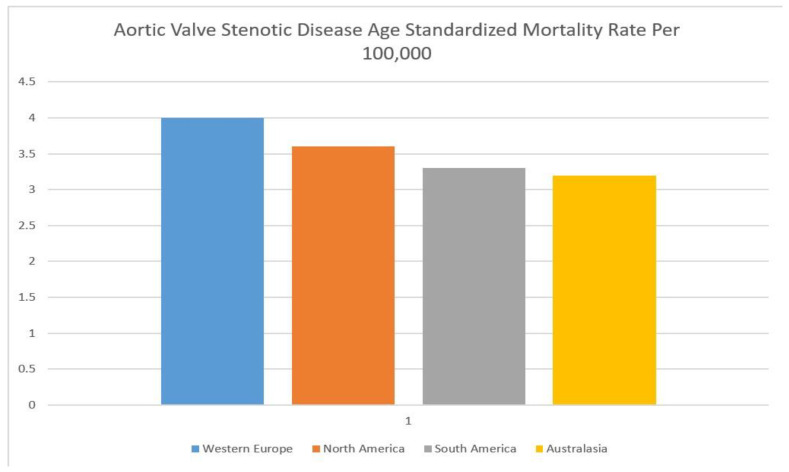
Bar chart showing aortic valve stenotic disease age-standardized mortality rate per 100,000.

An estimated 9.4 million patients worldwide had calcific AVSD in 2019 [1,2]. Rates of AVSD are highly correlated with old age and are the greatest in North America, Europe, and Australasia. The age-standardized AVSD mortality rate per 100,000 people is highest in Western Europe (4.0, 95% CI 3.4–4.5), North America (3.6, 95% CI 3.0–4.0), South America (3.3, 95% CI 2.9–3.7), and Australasia (3.2, 95% CI 2.6–3.6) (Figure 2) [10]. These regions have the highest rates of common AVSD risk factors such as hypertension, hyperlipidemia, and obesity and a greater life expectancy than countries in the developing world. A lack of access to echocardiography among developing nations may also explain the discrepancies in the reported AVSD rates.

Rheumatic aortic stenosis is most common in Asia, with a reported prevalence of 4.54 in India, 1.86 in China, and 1.3 in Bangladesh per 1000 people. Several Asian countries, including Singapore, Japan, and South Korea mirror Western cohorts in having a very low prevalence of rheumatic valvular disease. The prevalence of rheumatic aortic stenosis is 0.4 in Singapore, 0.14 in Japan, and 0.5 in South Korea per 1000 people. Singapore, Japan, and South Korea have a high proportion of elderly citizens with degenerative aortic stenosis rather than rheumatic aortic stenosis [11].

Several studies have shown that the prevalence of severe aortic stenosis is lower in African Americans than Caucasians [12].

AVSD was estimated to be responsible for approximately 127,000 global deaths in 2019 and an associated loss of 1.8 million DALYs (disability-adjusted life-years) [1,2]. According to a large Australian registry, the 5-year mortality rate is 56% for moderate AVD and 67% for severe AVSD [13]. Global deaths have increased by 138% between 1990 and 2019 likely due to global population aging and the “Westernization” of lifestyle, with the greatest increases having been observed in transitioning economies like China [10].

Mortality rates have decreased in developed countries with the advent of new interventions, though no therapies are currently available to prevent the disease. Intervention is indicated once the ejection fraction (EF) falls below 50% or if the patient is symptomatic. There is variation in the geometric ventricular patterns that are present in severe ASVD, with patients with “mixed” hypertrophy being at increased risk of developing symptoms [14]. Cardiac catheterization is first required in order to rule out coronary artery disease. Without treatment, the prognosis is poor, with the 5-year mortality rate increasing to 94% for those who cannot tolerate surgical or transcatheter intervention (TAVR) [15]; however, one large US study suggested that there is no significant survival difference between these two interventions [16]. Patients undergoing TAVR who retain a high transvalvular gradient, however, have been shown to ultimately suffer a poorer prognosis [17].

### 2.2. Risk Factors

While the bicuspid aortic valve is the most common form of congenital heart disease, occurring in 1–2% of the general population, it accounts for the presentation of 25% of patients >80 years who are referred for aortic valve replacement and 10% of patients who are undergoing TAVR [18]. Bicuspid aortic valve is more prevalent among patients with Turner’s syndrome and other rare congenital diseases, with a prevalence of 6.4% among first-degree relatives with the condition, suggesting familial clustering [19]. While bicuspid valve patients undergoing TAVR with early-generation devices had lower success rates, the operative success rates are not significantly different for newer-generation models [20]. Early screening and surgical intervention has been proposed for bicuspid valve patients, though there is no current standard of care [21]. Congenital bicuspid aortic valves are found in a lower frequency in African Americans. In Asian populations, a higher prevalence of bicuspid aortic valve (BAV) is seen in Chinese patients. Type 1 BAV with raphe between the right and noncoronary cusps is more commonly seen in Asian patients than European patients and they also experience less type 0 BAV that is no-raphe [22].

Smoking is a significant risk factor for AVSD; one case-control study established an odds ratio of 5.3 to 1 for significant AVD among all smokers and 10.0 to 1 among smokers with >40 pack-years, suggesting a dose-dependent relationship. Former smokers who had quit >10 years ago had a significantly lower risk than current smokers [23].

Hypertension is the strongest modifiable risk factor for AVSD, though effective hypertension management can be complicated by severe stenosis. ACE inhibitors are the best-studied antihypertensives for AVSD patients. The most effective means of hypertension prevention include the Dietary Approaches to Stop Hypertension (DASH) diet and weight loss [24].

Chemotherapy and radiation therapy to the chest increase the risk of valvular heart disease. Specifically, a case-control study of Hodgkin’s lymphoma patients found a dose-dependent increased risk from 1.4–11.8 depending on the radiation dose (<30–>40 Gy), with the aortic valve being that which was most frequently affected. Today, mediastinal radiation doses are typically 20–30 Gy, which increase the 30-year valve disease rates by only 3% [25]. Exposure to anthracycline-based chemotherapy was shown to increase the risk of valvular heart disease (HR = 1.5) and heart failure (HR = 3.0) in Hodgkin’s disease patients independent of radiation, likely due to cardiac remodeling [26].

## 3. Rheumatic Heart Disease

### 3.1. Epidemiology

Rheumatic heart disease (RHD) remains the most common cause of valvular disease worldwide. Data on RHD from hospital registers includes predominantly moderate–severe RHD, suggesting the true prevalence of RHD (mostly mild cases) is likely much higher than is characterized. Mortality from RHD is multifactorial, with RHD predisposing to atrial fibrillation (which increases the risk for stroke), as well as heart failure, pulmonary edema, and cardiorenal syndrome [27].

RHD follows pharyngitis by group A Streptococcus (Streptococcus pyogenes), resulting from molecular mimicry between the bacterial antigens and cardiac “M-protein”. RHD most commonly affects the mitral and aortic valves, presenting in children and young adults (who are at the highest risk of streptococcal pharyngitis) with regurgitation. In contrast, older adults tend to present with stenosis due to a decade-long process of commissural fusion, valvular fibrosis, and ultimate calcification. Mitral stenosis typically presents with a long asymptomatic period followed by gradual dyspnea on exertion and findings of right heart failure and pulmonary hypertension. The normal mitral valve area is 4–6 cm^2^; severe mitral stenosis occurs when the mitral valve is less than 1 cm^2^ in area. Late findings include hemoptysis that is secondary to pulmonary hypertension and thromboembolic stroke, as well as hoarseness due to the compression of the laryngeal nerve and dysphagia due to the compression of the esophagus by an enlarging left atrium [28].

RHD was estimated to affect 40.5 million people worldwide in 2019, with an annual incidence rate of 2.8 million. It also accounted for an estimated 306,000 global deaths, with a median age of 28.7. Because it affects younger adults, an estimated 8.7 million life-years and 10.7 DALYs are lost to RHD each year [1,2]. The highest mortality rates for RHD are observed in Oceania, South Asia, and sub-Saharan Africa. The global RHD prevalence continues to increase annually, although the age-standardized prevalence has increased much slower, reflecting the predomination of growing, young populations. RHD prevalence has also increased faster than its incidence rate, reflecting a reduction in premature mortality. Of note, in every year since 1996, India has set and surpassed the world record for RHD cases and deaths. Nonetheless, globally, age-standardized RHD mortality per 100,000 people fell from 9.2 in 1990 to 4.8 in 2015 [29].

### 3.2. Risk Factors

The lack of access to antibiotics, overcrowding, and poor hygiene that are associated with poverty are the predominant risk factors for RHD. It is more prevalent in the Indian subcontinent, in sub-Saharan Africa, the Middle East, and in certain areas of South America. Increased prevalence has been seen in Oceania, especially among the indigenous populations of New Zealand and Australia. Improved living conditions in transitioning nations, such as China and Russia, have led to decreasing national rates. However, impoverished populations in those nations, such as their respective indigenous groups, continue to suffer high rates of RHD [1,2]. Similarly increased risk is seen among indigenous groups in developed nations like Australia, New Zealand, and the US [30,31,32]. Public health initiatives like the Rheumatic Fever Prevention Program in New Zealand, which focuses on improving disease awareness, reducing crowding, and administering antibiotics in a timely fashion among indigenous groups that are at heightened risk, have not been successful, resulting in no overall improvement in hospitalization between 2012 and 2017 [33]. Research is currently focused on developing a group A streptococcus vaccine to reduce the growing global RHD burden.

Rheumatic fever does not have a markedly different incidence rate between the sexes, although certain clinical manifestations, such as Sydenham chorea and mitral stenosis, are more common in females who have gone through puberty. Acute rheumatic fever is relatively rare in infants and uncommon in preschool-aged children. It is most common among children who are aged 5–15 years. Acute rheumatic fever occurs in young adults as well, but the incidence rate of the occurrence of a first episode of rheumatic fever falls steadily after adolescence and it is rare after age 35 years. Decreased risk of streptococcal infections in adults may be the cause of the lower rate of acute Rheumatic fever in this population cohort. Recurrent episodes, with their predisposition to cause or exacerbate valvular damage, occur until middle age.

## 4. Mitral Regurgitation

### 4.1. Epidemiology

Mitral regurgitation (MR) is the third most common form of valvular heart disease, affecting approximately 24.2 million people around the world. Since MR is largely a disease of older adults, it has resulted in an estimated 0.88 million DALY and 34,000 deaths in 2019 [1,2]. MR is either equally prevalent between the sexes or slightly more prevalent in men than women. Mitral valve prolapse rates (MVP) are considerably higher in white patients than in black patients [34].

Whether primary or degenerative, MR is most commonly a sequela of myxomatous degeneration and mitral valve prolapse. MVP is the most common cardiac mitral valvular pathology worldwide, accounting for 2% to 3% of the total population. RHD remains prevalent in developing countries and is the most common cause of mitral valvular pathology resulting in hospital admissions. Acute MR can present with sudden onset of dyspnea and flash pulmonary edema, frequently secondary to myocardial infarction. Chronic MR is often asymptomatic, though treatment is recommended prior to symptom onset. Physical exam reveals a soft S1 and a holosystolic, bowing murmur optimally appreciated at the cardiac apex with radiation to the axilla (in contrast to aortic stenosis, which radiates to the carotids). The presence of S3 suggests severe MR and impending systolic heart failure. The prevalence of primary MR has increased by 70% from 1990 to 2017, largely in developing nations, though age-standardized prevalence has not changed significantly, and mortality has fallen by about 32% [1,2].

### 4.2. Risk Factors

MVP, which underlies primary MR, is the most commonly diagnosed valvular heart disease, affecting an estimated 3–5% of the general population [35]. It can predispose an individual to MR, arrhythmias, endocarditis, and stroke [36]. MVP is an abnormally thick, redundant mitral valve leaflet than displaces in the left atrium during systole. MVP is often congenital and associated with connective tissue disorders such as Marfan syndrome, but it can also develop or exacerbate with hyperthyroidism, pregnancy, and other high-flow states. MVP may be complicated by a chordal rupture or endocarditis, both of which can lead to severe MR. MVP is often appreciated as a midsystolic click followed by a midsystolic murmur that increases in intensity with handgrip or a Valsalva maneuver [36,37,38]. Degenerative MR demonstrates less regional variation than RHD or ASVD as it is associated with neither poor living conditions nor atherosclerosis [10]. Improved access to echocardiography will likely result in an increased incidence in the developing world, while access to surgical and transcatheter interventions will continue to decrease mortality rates [1,2].

Secondary, or functional, MR accounts for 65% of cases of moderate–severe MR and affects 24% of patients with systolic congestive heart failure (CHF). It results from the morphological dilation of the left atrium or ventricle with no change to the mitral valve leaflets. Secondary MR is most common in developed nations with high rates of atherosclerosis, coronary artery disease (CAD), and CHF, all of which predispose to, and are associated with, secondary MR. Surgical and transcatheter interventions for secondary MR are controversial, though the recent trial of transcatheter mitral valve repair in patients with heart failure (COAPT) did demonstrate a prognostic benefit of the intervention. The outcomes are best in patients with an EF > 60% and a LV end-systolic diameter of less than 4.5 cm [39]. MitraClip is a procedure that is reserved for patients with primary degenerative MR who are at too high a risk for surgery. For patients with EF under 30%, medical management and/or a left ventricle assist devices are evidence-based options.

## 5. Aortic Regurgitation

### 5.1. Epidemiology

Aortic regurgitation (AR) is the fourth most common valvular disease in the world [1,2]. Although global estimates are unavailable, AR was detected in 1.6% of UK elders aged > 65 years [40], 1.8% of Swedes aged > 65 years old [41], and 1.1% of Chinese citizens aged > 60 years old [42]. In contrast, 4.9% of participants in the US-based prospective Framingham study had AR detected, though only 0.5% of them had moderate–severe disease [43]. These discrepant statistics imply that mild disease is likely under-detected in the general population, especially in resource-poor developing nations with limited access to echocardiography. Data on the AR rates in these nations are largely unavailable. The age-standardized incidence of AR in Denmark increased from 2000 to 2017, similar to ASVD [44], though, unlike ASVD, AR is not known to be significantly associated with atherosclerosis. Acute AR can present with rapid cardiogenic shock, while chronic AR has a long asymptomatic period followed by gradual, progressive dyspnea. A physical exam will reveal a soft or nonexistent A2 and a decrescendo blowing diastolic murmur that is best heard at the cardiac base, as well as a wide pulse pressure, which is often described as a water hammer peripheral pulse or a nail bed pulsation (Quincke pulse). One can also appreciate a popliteal brachial blood pressure difference of greater than 20 mm Hg.

### 5.2. Risk Factors

AR can result from primary valve pathologies, such as BAV, connective tissue disease, RHD, infective endocarditis or autoimmune disease, or as a secondary pathology to aortic root dilation. On top of their significant risk of ASVD, 30% of patients with a bicuspid valve are diagnosed with moderate–severe AR on their first presentation. Interestingly, women with bicuspid valves are more likely to develop AR while men are more likely to develop ASVD [45]. Patients with RHD are likely to develop mild AR only in tandem with mitral valve disease. As for secondary AR, the aortic root dilation is most often associated with diastolic, but not systolic, essential hypertension and the risk increases with age [46].

The etiologies that are responsible for chronic AR may be extensive, they include rheumatic heart disease (the most common cause in the developing world), IE, myxomatous valve degeneration, congenital valve abnormalities, age-related dilatation of the aorta, aortic dissection, aortitis/aortic root dilatation secondary to syphilis or giant cell arteritis, trauma, systemic hypertension, senile valvular calcifications, drug-induced valvulopathy, ectasia of the aortic annulus, Crohn disease, Whipple disease, and osteogenesis imperfecta [47]. Recent advances in the repair of aortic dissections have resulted in lower levels of mortality and a reduced risk of complications like AR [48].

The other rheumatologic processes that are involved are systemic lupus erythematosus, rheumatoid arthritis, antiphospholipid syndrome, Reiter syndrome, ankylosing spondylitis, psoriatic arthritis, Takayasu vasculitis, relapsing polychondritis, Ehlers–Danlos syndrome, Behçet disease, and Marfan syndrome.

Aortic root dilation is also known to have a strong genetic component, based on twin studies [49], and to be associated with inherited pathologies such as homocystinuria [50], Marfan syndrome, and Turner’s syndrome [51]. Correlations have also been made between Turner’s syndrome and AR. In a study of 253 subjects with Turner’s syndrome who were aged between 7 to 67 years (with 89 subjects being less than 18 years old), AR was trivial or less in 55%, mild in 30%, and moderate to severe in approximately 15% of the studied population, respectively [52].

## 6. Tricuspid Regurgitation

### 6.1. Epidemiology

Tricuspid regurgitation (TR) is the least common primary valvular pathology, although it is associated with significantly increased mortality (up to 42% in 3 years, in one study) [53]. TR typically presents with symptoms of isolated right heart failure, such as jugular venous distension, hepatojugular reflex, peripheral edema, and ascites. No global data is available, though national screening studies have revealed disparate prevalence, with 2.7% of older individuals showing moderate–severe TR in the UK [40] and only 1.1% of similar-age patients in China showing TR [42]. A US community cohort found a prevalence of only 0.55% [54]. A study from the US from 2008 to 2018 revealed the age-adjusted mortality due to TR to be unchanged until 2013, after which the mortality rate began to increase by about 25% per year [55]. These findings may be attributable to the increased incidence of HF and subsequent use of intracardiac devices [56].

### 6.2. Risk Factors

TR arises largely secondarily to right ventricle or atrial dilation, with one US study reporting that 92% of cases were associated with another cardiac pathology, most commonly left heart failure [54]. Nonetheless, TR is an independent risk factor for morbidity and mortality, even in cases of moderate disease [54]. TR is a feared complication of intracardiac pacemaker placement, with patients with underlying right ventricle dilation suffering an increased risk and poorer prognosis following placement [56]. TR is also classically associated with infective endocarditis from Staphylococcus aureus, a condition which is common in IV drug users.

TR is rarely associated with carcinoid syndrome due to neuroendocrine tumors of the GI tract. Other symptoms may include flushing, diarrhea, wheezing, pellagra (Vitamin B3 or niacin deficiency due to the excess consumption of tryptophan), cognitive impairment, and other right heart valve defects due to the excess production of serotonin (5-HT) by the tumor; the left heart is not affected as serotonin is inactivated in the lungs. Carcinoid syndrome is diagnosed by elevated urinary 5-HIAA, a byproduct of serotonin metabolism, and treated with formulations of somatostatin [57].

Tricuspid regurgitation that is secondary to rheumatic heart disease is usually associated with aortic and mitral valve pathology. Deformation of the leaflets due to rheumatic disease is the most common cause of pure TR. The congenital malformation of the tricuspid valve is seen in Ebstein anomaly which is characterized by the apical displacement of the annular insertion of the posterior and septal leaflets and atrialization of a portion of the ventricular myocardium [58].

Secondary or functional TR can occur mainly due to left-sided pathology with pulmonary hypertension (mitral valve and aortic valve pathology and left ventricular pathology), right-sided pathology with pulmonary hypertension (idiopathic pulmonary hypertension, acute, or chronic lung disease), and global or regional right ventricular dysfunction (right ventricular ischemia, arrhythmogenic right ventricular cardiomyopathy, and sarcoidosis). Isolated TR can be seen in patients with atrial fibrillation [59,60].

## 7. Infective Endocarditis

### 7.1. Epidemiology

There were an estimated 1.1 million global cases of infective endocarditis (IE) in 2019, claiming approximately 66,000 lives and resulting in the loss of 1.7 million DALYs [1,2]. Developed nations have the greatest age-standardized prevalence, though the incidence rate varies drastically within and between countries, from a low of 5.7 annual cases/100,000 to a high of 35.8/100,000. IE is among the most acute deadly valvular pathologies, with an in-hospital mortality rate of 22% and a 5-year mortality rate of 45% [61]. The global prevalence of IE has increased by 44% and the age-standardized incidence has increased by 39% since 1990, with the greatest growth having been found in transitioning nations like China which had an initially lower prevalence [1,2]. This increase may be explained by the increased access to cardiac imaging and microbiological testing. The age-standardized mortality rate has not changed significantly over time, with the latest estimate being around 0.9/100,000 in 2019 [1,2].

### 7.2. Risk Factors

IE is most commonly subacute and is associated with Viridans streptococci species, although culture-negative endocarditis accounts for an approximate 30% of patients—largely due to the use of antibiotics before sampling, and it is rarely due to “HACEK” organisms (Haemophilus parainfluenzae, Haemophilus aphrophilus, Haemophilus paraphrophilus, Actinobacillus actinomycetemcomitans, Cardiobacterium hominis, Eikenella corrodens, and Kingella species) [62]. Other classical associations include Enterococcal IE following GI and GU procedures, Staphylococcal IE of the tricuspid valve among IV drug users, Coxiella and Brucella among farmers, and Streptococcus bovis IE in patients with underlying colorectal cancer. Organism-specific therapy is considered to be the gold standard [63]. Non-bacterial thrombotic endocarditis, which is associated with systemic lupus erythematosus, granulomatosis with polyangiitis, antiphospholipid antibody syndrome, Behçet disease, adult-onset Still disease, or malignancy-related (carcinoid) endocarditis, accounts for only 2.2% of the patients with culture-negative endocarditis [64].

The growth in the rates of IE in developed and transitioning nations is likely secondary to the prolonged life expectancy, increased prevalence of heart disease, higher number of patients with intracardiac devices and prosthetic valves, and higher rates of IV drug use. The incidence rate has particularly increased in the past 10 years, a change which has likely been catalyzed by the opioid addiction epidemic in the US and other developed nations, the emergence of Staphylococcus and Enterococcus as the predominant causative organisms, and greater rates of diagnosis of the condition [65]. Widespread antibiotic prophylaxis for IE has been challenged but is still recommended before dental procedures in select patients, such as those with prosthetic heart valves and congenital heart defects [66].

## 8. Conclusions

Valvular heart disease is a growing cause of global cardiovascular morbidity and mortality with a very disparate geographic distribution. Developed nations have seen a rise in pathologies of the aortic valve and MR as their populations grow older and chronic hypertension, atherosclerosis, and other forms of cardiovascular disease become more prevalent. Meanwhile, developing nations have seen an increased prevalence of RHD as their young-adult populations expand and premature mortality decreases with the greater availability of antibiotics, microbiological testing, and echocardiography. These pathologies are also overrepresented in the indigenous and impoverished populations of developed nations, leading to public health initiatives that are geared towards addressing these disparities. Meanwhile, TR and IE have grown more prevalent in developed nations with the advent and growing usage of intracardiac pacemakers and prosthetic valves, respectively. The burden of disease is likely to rise as diagnostic tools become more available in developing nations and as transitioning economies, such as China, adopt a more sedentary lifestyle and “Western” diet, thus increasing the global prevalence of cardiovascular disease. Primary care preventative guidance and screening, public health initiatives, improved living conditions, and earlier transcatheter valvular interventions will be key in stemming the growing burden of valvular heart disease.
